# A Review of COVID-19 Hotels in New York City During the Early 2020 Outbreak: An Alternative Care Model

**DOI:** 10.7759/cureus.64736

**Published:** 2024-07-17

**Authors:** Megan Krentsa, Katherine McCann, Heather Papowitz, Rituparna Pati, Tyler B Evans

**Affiliations:** 1 Medicine, Drexel University College of Medicine, Philadelphia, USA; 2 Population and Family Health, Columbia University Mailman School of Public Health, New York City, USA; 3 COVID-19 Response, World Health Organization, Geneva, CHE; 4 COVID-19 Response, World Health Organization, Atlanta, USA; 5 Population and Public Health Sciences, University of Southern California Keck School of Medicine, Los Angeles, USA

**Keywords:** covid-19, infectious disease control, public health system, urban health services, social determinants of health (sdoh), health disparities and vulnerable populations, coronavirus quarantine, covid-19 isolation, disease outbreak, community medicine & public health

## Abstract

New York City (NYC) was the epicenter of the early US COVID-19 pandemic. From March to May 2020, overburdened healthcare centers precipitated an emergent need for non-traditional facilities to meet patient care demands. Given travel restrictions and NYC's underutilized tourist infrastructure, hotels were available to support emergency response needs. This article describes the process by which NYC's non-medical COVID-19 hotel programs were selected, mobilized, and operated, including lessons learned.

NYC agencies and organizations collaborated, creating an interagency initiative that activated hotels to provide safe isolation and quarantine spaces for those diagnosed with or exposed to COVID-19, aiming to reduce community spread, increase capacity for NYC's strained healthcare system, and mitigate interagency redundancy. Interagency groups addressed hotel challenges, including infection prevention and control; behavioral health, intellectual, and developmental disorders; social determinants of health; and coordination, operations, and planning.

NYC's COVID-19 hotel program successfully supported overburdened hospitals by providing alternate locations for non-inpatient COVID-19 individuals. Community engagement required a methodical approach, balancing quality assurance with efficient access. An interagency coordinating body developed and shared clinical criteria for hotel admissions, infection prevention and control (IPC) procedures, and discharge plans, enhancing the program's ability to scale and address complex needs. Lessons learned from this program can be applied for smoother implementation of similar programs in the future.

## Introduction and background

In early 2020, New York City (NYC) was the epicenter of the coronavirus disease 2019 (COVID-19) outbreak, prompting the city to design new systems to contain the virus outside of traditional healthcare settings. The city developed an interagency approach to activate alternate care sites and COVID-19 hotels for isolation of persons with COVID-19 or quarantine of their contacts to mitigate the disease burden on the conventional health system. Several city agencies established hotel programs to address the needs of vulnerable populations who would otherwise lack a safe place to isolate or quarantine following a diagnosis with or exposure to COVID-19.

The city's response to the early surge of COVID-19 cases and deaths in NYC required novel strategies and innovative solutions. Like other cities, NYC was not prepared for the ways in which the outbreak would impact the city's most vulnerable populations. The interagency NYC COVID-19 hotel operations reflect one strategy that served the city's population during a public health emergency. This manuscript describes the mobilization of NYC's hotel program and the lessons learned from this experience.

## Review

Why hotels?

As the medical community began to understand SARS-CoV-2 progression and transmission modalities, it became clear that operationalizing hotels across NYC for isolation and quarantine was a logical approach to relieve pressure on the city's overwhelmed hospital systems and congregate care settings for the reasons outlined below.

NYC Tourist Infrastructure

NYC's tourist infrastructure is internationally known. The suspension of tourism during the pandemic caused hotels to be largely vacant, and the hotel industry suffered significant revenue loss. Using hotels as non-traditional care settings allocated otherwise unused space to meet an urgent need while supporting the hotel industry.

Effective Isolation

**Figure 1:**
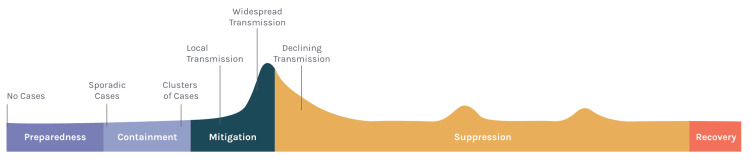
Phases of pandemic response and recovery as described by the prevent epidemics COVID-19 response playbook

Hotels provided space for the isolation of people with COVID-19 during the infectious period or quarantine of exposed individuals during incubation periods when SARS-COV-2 was most infectious, thereby preventing community spread (Figure 1).

Another deciding factor in the mobilization of hotels was the need for infection prevention and control (IPC) procedures to mitigate transmission. Facilities with opportunities for physical distancing - those with private bathrooms - were ideal solutions. Hotels could accommodate the NYS executive order for mandatory quarantine [1,2] for travelers arriving from international destinations with high rates of community transmission.

Isolating With Dignity

NYC prioritized the dignity and comfort of clients who needed to safely isolate and quarantine. Clients most at risk of adverse disease progression were those with chronic comorbidities and low health literacy. In hotels, clients could safely isolate in a dignified manner. Hotels provided safe environments for individuals experiencing housing insecurity, crowded living conditions (e.g., multigenerational homes), those whose families and/or landlords would not allow them to return due to fear of potential COVID-19 exposure, and those living in congregate care facilities.

The COVID-19 hotel landscape in NYC

Clients were directed to medical or non-medical hotels if they could not safely isolate or quarantine at home. This distinction was based on clients' clinical acuity, symptoms, and level of medical needs. Medical hotels were established by NYC Health and Hospitals (H+H) [3] to treat patients with acute medical needs from their COVID-19 diagnosis. H+H hotels provide a higher level of medical care, including around-the-clock clinical monitoring by medical personnel. This paper focuses on the contributions of the city's non-medical hotels.

Non-Medical COVID-19 Hotels

Non-medical hotels provided safe places for isolation and quarantine for individuals diagnosed with or showing symptoms of COVID-19, as well as their close contacts.

Hotels in both categories were operated by these city agencies: New York City Emergency Management (NYCEM) [4]; NYC Health and Hospitals (H+H) [3]; Department of Youth and Community Development (DYCD) [5]; Department of Homeless Services (DHS) [6]; and Mayor's Office of Criminal Justice (MOCJ) [7].

This paper describes the coordination and operations of the four types of non-medical hotels: isolation, quarantine, risk reduction, and healthcare personnel hotels.

Isolation Hotels

Isolation hotels provided a safe space for the isolation of individuals who were symptomatic with mild COVID-19-like illness or diagnosed with COVID-19. Some individuals were unable to safely isolate in their own residence because of their household type (e.g., multigenerational) or housing insecurity. Isolation hotels provided food delivery and laundry services, thereby minimizing contact with others. Isolation hotels offered clinical assessments and support services. This comfortable and dignified isolation experience increased client compliance with public health recommendations.

Quarantine Hotels

Quarantine hotels served individuals who were in close contact with someone with COVID-19, offering many similar services to isolation hotels.

Risk Reduction Hotels

Risk-reduction hotels served individuals without COVID-19 or COVID-19 symptoms who were at heightened risk based on residence, employment, or lifestyle. Examples include people living in congregate settings (e.g., supportive housing, homeless shelters, correctional facilities, universities), people experiencing housing insecurity, and people with behavioral health needs or substance use disorders (SUD) impacting their ability to follow IPC measures.

Some risk-reduction programs focused solely on reducing the risk of SARS-CoV-2 transmission, such as density reduction or decongestion of facilities with high structural and demographic risk factors. Other programs had additional policy objectives, such as the Mayor's Office of Criminal Justice (MOCJ) program that provided housing to clients who were granted early release from prison. Hotels provided a safer space - with personal rooms and bathrooms, support services, meals, and IPC practices - to decrease COVID-19 exposure risk.

Healthcare Personnel Hotels

Healthcare personnel (HCP) hotels provided temporary housing for front-line workers who were at increased risk of COVID-19 infection. These hotels reduced potential exposure to household members should HCP become infected via occupational exposure.

COVID-19 hotel functions

Hotel programs fulfilled public health objectives by mitigating SARS-CoV-2 transmission and offloading pressure from the conventional healthcare system and congregate care facilities and providing a safe place for HCPs to rest if they were concerned about transmitting COVID-19 to household members.

A successful hotel program achieves the following: provides outreach and eligibility screening to ensure that populations needing care can access the hotel or are referred to an appropriate alternate location; provides client-focused clinical support that considers Social Determinants of Health (SDoH); and installs a robust IPC framework of equipment, training, and access control procedures to minimize infection risk for program staff and clients.

**Figure 2:**
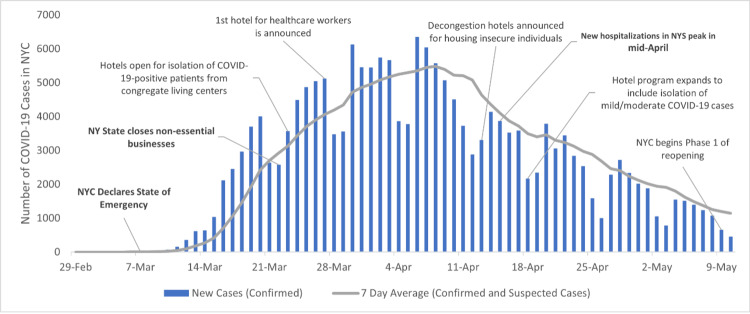
Timeline of NYC COVID-19 hotel program development in spring 2020 NYC - New York City

As community transmission rapidly increased (Figure 2), mandatory quarantine gave way to mitigation to manage the high caseload in overwhelmed NYC hospitals. During this phase, the city's COVID-19 hotels aimed to reduce strain on the healthcare system. Hotels provided an alternate site for individuals with no or mild-to-moderate symptoms and those who were recovering from their hospital stay but who still needed to complete the duration of isolation. By providing a safe place for individuals to isolate and quarantine during the period of peak transmissibility, community spread decreased.

NYC COVID-19 hotel interagency coordination initiative

During the first wave of COVID-19 infections, different city agencies, some of which are listed below, activated hotels with context-specific modifications to address client and programmatic needs. It became clear that many agencies were operating similar programs. Therefore, to mitigate redundancy and build capacity, the New York City Emergency Management (NYCEM) and the NYC Department of Health and Mental Hygiene (DOHMH) convened the Interagency Coordination Initiative to standardize operations across hotel programs. Several agencies joined the collaborative effort, including the NYC Department of Health and Mental Hygiene (DOHMH) [8]; the Office of the Mayor of New York City [9]; Hudson River Health (HRH) (now Sun River Health) [10]; Housing Works [11]; Médecins Sans Frontières (MSF)/Doctors Without Borders [12]; and the Office for People With Developmental Disabilities (OPWDD) [13]

The Initiative identified policy objectives and generated working groups with subject matter experts (SMEs) in their fields, standardizing an approach to common issues encountered at hotels. Those groups were as follows:

Infection Prevention and Control (IPC)

The IPC group convened interagency infection control SMEs to share evidence-based recommendations and standardize best practices to prevent SARS-CoV-2 transmission within participating hotels. This group performed on-site hotel evaluations and developed tools, protocols, and guidance to optimize IPC measures while training staff on best practices. This group's guidance [14] was shared with community partners who supported hotel operations.

Behavioral Health, Intellectual, and Developmental Disorders (BH/I/DD)

The BH/I/DD interagency group developed and implemented recommendations for hotel clients with mental illness, substance use disorders (SUDs), and I/DD who needed help with activities of daily living (ADLs). Vulnerable populations faced unique isolation and quarantine challenges, and the BH/I/DD group reduced barriers to isolation by designing guidance for engaging, comfortable, and supportive environments for hotel clients.

Social Determinants of Health (SDoH)/Social Services Delivery

The SDoH interagency group convened SMEs and community-based organizations (CBOs) to develop guidance for accessing human services, especially when SDoH barriers might impair the client's ability to isolate, quarantine, and maintain health after hotel check-out. The pandemic placed extraordinary pressure on NYC's social support systems, disproportionately impacting communities of color. SDoH risk factors emerged as vulnerabilities for many New Yorkers, increasing the likelihood of exposure and susceptibility to adverse sequelae of COVID-19. Housing instability, unstable income, and risk of job loss emerged as barriers to isolation and quarantine for vulnerable communities, and these became hotel discharge considerations. NYC agencies launched a just-in-time gap analysis group to understand the resources available to New Yorkers who needed to isolate or quarantine at home or in hotels. The gap analysis informed public health officials about city-wide strategies to reduce SARS-CoV-2 transmission and mitigate disease impact.

Coordination, Operations, and Planning

The Coordination, Operations, and Planning Group consisted of senior members of NYC agencies with response operations experience. Its core objective was to provide high-level direction and implement hotel policies and recommendations. This group provided insight into the feasibility of program recommendations and contextualized guidance for each hotel type based on its population's needs.

COVID-19 hotel mobilization and operations

Hotel Mobilization Criteria

Hotels were selected based on location and specifications. Location considerations included proximity to overburdened healthcare facilities or communities with a high demand for isolation and quarantine services. Once a potential location was identified, a stand-up team deployed for a site review, evaluating the building's infrastructure, floor plan, entrances and exits, and safety features. The stand-up team's checklist determined if a site was appropriate for use. New hotels were established when previous sites reached 75% occupancy.

Infection Prevention and Control (IPC)

IPC is a critical component of a successful isolation or quarantine hotel program. Standard IPC practices were adapted to fit the hotel environment's needs, and every effort was made to protect clients and staff, including promoting self-monitoring and reporting of COVID-19 symptoms. From screening individuals via temperature and symptom checks at hotel entrances to limiting the number of passengers in elevators, all locations within the hotel were assessed and monitored to reduce exposure risks. Key features of floor plans that limit cross-contamination within healthcare facilities were replicated if possible, in hotels, such as the use of clean and soiled utility rooms, separate spaces for personal protective equipment (PPE) donning and doffing, and dedicated areas for administrative work and staff breaks. Signs posted throughout hotels promoted hand hygiene, physical distancing, and mask use. Protocols standardized best practices for housekeeping, linen management, food services, and environmental cleaning and disinfection. Staff received training to ensure familiarity with IPC processes.

Organizational Structure and Staffing

**Figure 3:**
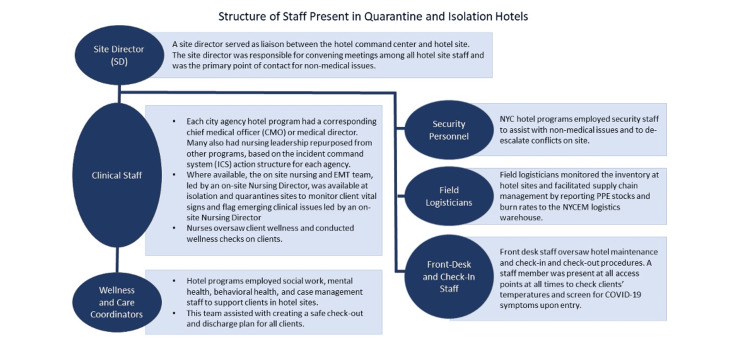
General structure of staff in most quarantine and isolation hotels PPE - personal protective equipment; NYC - New York City; NYCEM - New York City Emergency Management

A hotel command center with representatives from city agencies set policy, provided logistical support, and contracted staff to operationalize isolation/quarantine hotel sites. The staff at sites differed according to client needs, but most sites employed staff in the roles listed in Figure 3. Front-desk staff were often existing hotel staff who were familiar with the building.

Hotel staff received instructions for incident reporting and HIPAA-compliant transfer of patient electronic health records. Clinical staff transferred hotel clients whose clinical presentation deteriorated during their stay to hospitals.

Community, Partner, and Provider Outreach and Engagement

Outreach and engagement occurred at several levels, from healthcare providers, CBOs, community boards, congregate settings partners and providers, and associations such as the Greater New York Hospital Association (GNYHA) and the Community Healthcare Association of New York State (CHCANYS). H+H also provided a presentation to its 100+ One City Health Medicaid redesign partners and intergovernmental agencies, such as the Department of Consumer and Worker Protection and H+H's affiliated CBOs - H+H's Test&Trace [15]. Resource Navigators performed outreach to provide information on the hotel program.

Referrals

The referral process was critical to ensuring an effective program. In collaboration with the NYC DOHMH, NYCEM and H+H accepted clients by balancing clinical appropriateness with access. Federally Qualified Health Centers (FQHCs) and supportive housing networks connected with underserved populations directed appropriate clients to hotels.

Client Screening/Triage

Isolation and quarantine hotels had inclusionary and exclusionary criteria to ensure that individuals could safely shelter with minimal oversight. The criteria included mortality risk, ability to care for and isolate at home, ability to complete activities of daily living (ADLs), and other mental and behavioral health measures. The intake process began with a registered nurse who received information from the central screening unit's phone line before client arrival. Clients requiring higher levels of care were referred to more acute H+H medical hotel programs or hospitals.

Client Intake

Upon entry, clients were supported through check-in and medical and social work evaluations. At intake, individualized wellness plans were created, and the discharge planning process began to address social determinants of health.

Wellness Checks

Clients interacted with staff at least twice daily to assess client safety. During checks, clinical staff evaluated vital signs and offered social service connections. Clinical staff evaluated clients' risk of COVID-19 disease progression. Each client's individualized wellness plan determined the frequency and content of checks. Clinical (nursing) staff was on site 24/7, and physicians were on-call for emergencies.

Medication Management

Non-medical hotels provided minor clinical supportive services and basic first aid. Clients brought a 14-day supply of their own medications for self-administration. Medication-assisted treatment (MAT) for SUD and life-saving medications were exceptions, and clinical staff kept these on-site. NYC created a methadone delivery system for hotels (led by NYC DOHMH) and a virtual buprenorphine clinic (led by H+H).

Hospitality Services

Clients in hotels were provided food that met dietary preferences, laundry services, and access to Wi-Fi, phones, and a television. On-site staff connected clients to remote support groups.

Hotel Check-Out

Following then-current CDC recommendations, the default check-out date for isolation clients followed the below criteria: 10 days past symptom onset (or specimen collection date of positive test if asymptomatic, 14 days for discharge to a congregate setting); symptom improvement relative to baseline; and fever-free for 24 hours without use of fever-reducing medication

Deep cleaning of hotel rooms was performed by a third-party vendor after clients checked out.

Hotel Demobilization

Shutting down an isolation or quarantine hotel site took two weeks. On-site logistics personnel coordinated the safe check-out of guests and transferred clients requiring additional medical care. Once guests and equipment were removed, a third-party vendor completed building decontamination. Involved agencies convened an after-action report (AAR) to improve future programs.

Lessons learned

Identifying Hotel Purpose, Population, and Site

Identifying the hotel's purpose and how it fits with other programs within the city, state, or region is essential to ensure that programs are not duplicative and to prevent gaps in protection for vulnerable populations. Because different hotel categories seek to accomplish distinct goals, the intended purpose of the hotel program(s) and its target populations should be identified early to determine the best physical sites, equipment, staff, and IPC protocols. A methodical approach to engage vulnerable populations within communities is key. Intake pathways must balance quality assurance (appropriate clients) and efficient access.

Establishing Appropriate Clinical and Staff Support

Once a site and target population are selected, it is critical to assemble appropriate field and administrative support teams. A client intake system that considers SDoH and BH/I/DD needs is crucial to operating a safe, dignified, and effective program. This can be accomplished by establishing protocols for wellness checks and social service support. It is important to establish a center to respond to complex scenarios and incident reports.

Developing Defined Clinical Criteria for Hotel Admissions

Hotel programs must establish eligibility and exclusion criteria for their target population, specifying age restrictions and clinical acuity such as oxygen requirements, comorbidities, mobility level, and ADL independence. Programs must establish pathways for clients to access services, either through self-referral or healthcare provider referral. A logistics and operations team should procure equipment and organize transport with vendors.

Developing Defined IPC Procedures

Different hotels have distinct IPC needs. Staff should be trained on IPC procedures specific to each setting to ensure staff and client protection. Removing certain room doors and rearranging hotel furniture to allow for staff work areas, break rooms, client monitoring, personal protective equipment donning and doffing stations, and equipment storage are some examples of creative ways to utilize hotel space.

Interagency Cooperation

Interagency and city-wide cooperation is indispensable to the success of the use of hotels for public health purposes. Stakeholder agencies should maintain effective communication to ensure that the transfer of clients and information across hotel programs is transparent. Establishing a coordinated, interagency system builds capacity and integrates supplies and services among various hotel programs. Because of the unprecedented nature of the pandemic, individuals across agencies broke down organizational silos to collaborate, innovate and respond efficiently to the city's public health needs.

Hotel Discharge and Demobilization

Establishing checklists to ensure that clients are ready and safe for discharge into the community is key. To avoid unnecessary hospital transfers, clients should have housing accommodations and follow-up appointments scheduled. Early identification of hotel program demobilization procedures ensures a smooth demobilization process. Post-demobilization staff AARs produce informative reports to improve future iterations of public health hotel programs.

## Conclusions

The NYC COVID-19 hotel program improved throughout its lifecycle. A coordinated response between stakeholder agencies best served all New Yorkers by directing them to a safe isolation, quarantine, or risk reduction program based on their needs. However, this city-wide hotel program encountered initial challenges with staffing and streamlining resources while attempting to scale quickly enough to meet client needs. Establishing a coordinating body with standardized IPC protocols, inventory management plans, and staffing patterns enhanced the program's ability to scale up to more complex needs. Lessons learned from this program can be applied for smoother implementation of similar programs in the future.
